# Recapitulation of human pathophysiology and identification of forensic biomarkers in a translational model of chlorine inhalation injury

**DOI:** 10.1152/ajplung.00162.2023

**Published:** 2024-02-06

**Authors:** Satyanarayana Achanta, Michael A. Gentile, Carolyn J. Albert, Kevin A. Schulte, Brooke G. Pantazides, Brian S. Crow, Jennifer Quiñones-González, Jonas W. Perez, David A. Ford, Rakesh P. Patel, Thomas A. Blake, Michael D. Gunn, Sven E. Jordt

**Affiliations:** ^1^Department of Anesthesiology, Duke University School of Medicine, Durham, North Carolina, United States; ^2^Department of Biochemistry and Molecular Biology, Saint Louis University, St. Louis, Missouri, United States; ^3^Division of Laboratory Sciences, National Center for Environmental Health, Centers for Disease Control and Prevention, Atlanta, Georgia, United States; ^4^Center for Free Radical Biology and Lung Injury and Repair Center, The University of Alabama at Birmingham, Birmingham, Alabama, United States; ^5^Department of Medicine, Duke University School of Medicine, Durham, North Carolina, United States; ^6^Department of Pharmacology and Cancer Biology, Duke University School of Medicine, Durham, North Carolina, United States; ^7^Integrated Toxicology & Environmental Health Program, Duke University, Durham, North Carolina, United States

**Keywords:** acute lung injury, chlorine, medical countermeasure, pig translational model, US FDA animal rule (21 CFR 314.600)

## Abstract

Chlorine gas (Cl_2_) has been repeatedly used as a chemical weapon, first in World War I and most recently in Syria. Life-threatening Cl_2_ exposures frequently occur in domestic and occupational environments, and in transportation accidents. Modeling the human etiology of Cl_2_-induced acute lung injury (ALI), forensic biomarkers, and targeted countermeasures development have been hampered by inadequate large animal models. The objective of this study was to develop a translational model of Cl_2_-induced ALI in swine to understand toxico-pathophysiology and evaluate whether it is suitable for screening potential medical countermeasures and to identify biomarkers useful for forensic analysis. Specific pathogen-free Yorkshire swine (30–40 kg) of either sex were exposed to Cl_2_ (≤240 ppm for 1 h) or filtered air under anesthesia and controlled mechanical ventilation. Exposure to Cl_2_ resulted in severe hypoxia and hypoxemia, increased airway resistance and peak inspiratory pressure, and decreased dynamic lung compliance. Cl_2_ exposure resulted in increased total leucocyte and neutrophil counts in bronchoalveolar lavage fluid, vascular leakage, and pulmonary edema compared with the air-exposed group. The model recapitulated all three key histopathological features of human ALI, such as neutrophilic alveolitis, deposition of hyaline membranes, and formation of microthrombi. Free and lipid-bound 2-chlorofatty acids and chlorotyrosine-modified proteins (3-chloro-l-tyrosine and 3,5-dichloro-l-tyrosine) were detected in plasma and lung tissue after Cl_2_ exposure. In this study, we developed a translational swine model that recapitulates key features of human Cl_2_ inhalation injury and is suitable for testing medical countermeasures, and validated chlorinated fatty acids and protein adducts as biomarkers of Cl_2_ inhalation.

**NEW & NOTEWORTHY** We established a swine model of chlorine gas-induced acute lung injury that exhibits several features of human acute lung injury and is suitable for screening potential medical countermeasures. We validated chlorinated fatty acids and protein adducts in plasma and lung samples as forensic biomarkers of chlorine inhalation.

## Graphical abstract

**Figure:**
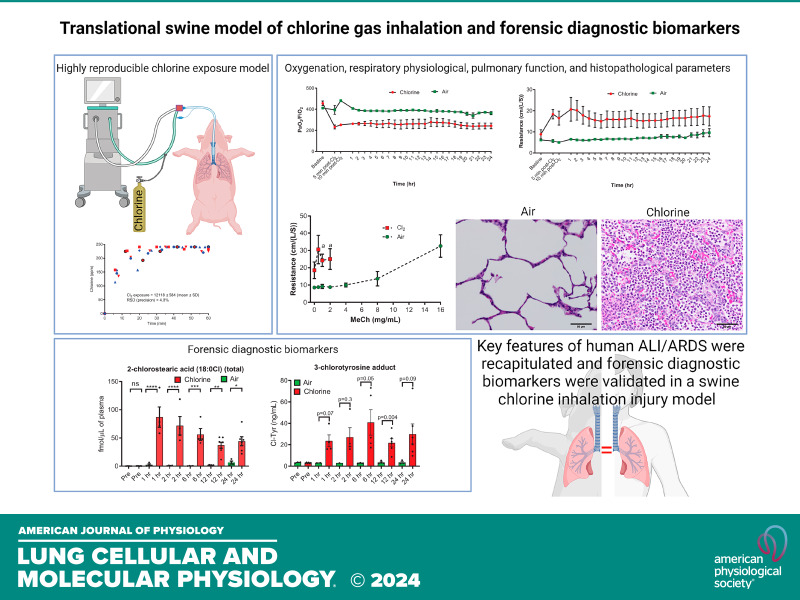

## INTRODUCTION

Chlorine gas (Cl_2_) is a highly reactive toxic halogen. More than 70 million tons of Cl_2_ are produced worldwide, for use in sanitation and the production of a wide range of chemicals. Cl_2_ has been used as a chemical weapon, first in World War I and most recently in Cl_2_ bomb attacks by the Syrian regime (1, 2), as proven by fact-finding missions of the Organization for the Prohibition of Chemical Weapons (1). Since Cl_2_ can be easily synthesized or diverted, there is rising concern about its potential use by terrorist groups.

It is estimated that Cl_2_ accounts for 84% of the total toxic inhalation hazards transported every year. Recent epidemiological studies after transportation accidents provided the most accurate descriptions of the morbidity and mortality associated with mass Cl_2_ exposures (3–7). The train derailment incident in Graniteville, SC, USA, in 2005 is considered one of the largest Cl_2_ accidents worldwide. In that incident, nine exposure victims died and 200 were admitted for inhalational injuries (3, 5, 6, 8). In addition to acute casualties, nonresolving chronic inflammation and airway hyperreactivity have been widespread in victims of this accident (6, 9, 10).

The pathophysiology and symptoms of acute lung injury (ALI) caused by Cl_2_ are highly variable, and depend on exposure concentration and duration (11–14). The common acute symptoms of Cl_2_ inhalation are consistent with acute respiratory impairment, and include running nose, sore throat, cough, choking, tightness of chest, labored breathing, bronchospasms, and wheezing (3, 13, 15). Exposure to higher concentrations of Cl_2_ for a prolonged period may result in noncardiogenic edema. At concentrations above 1,000 ppm, Cl_2_ can be fatal to humans within a few minutes (14, 16).

Due to increasing concerns about Cl_2_ as a potential chemical threat agent and propensity for mass casualties with accidental exposure or deliberate attacks, there is an immediate need for the development of therapeutics for Cl_2_-induced ALI. Several Cl_2_ plume models have also clearly illustrated the gravity of a Cl_2_ attack or accidental release (17–19). At this time, the standard of care only includes general supportive interventions such as humidified supplemental oxygenation, mechanical ventilator support, correction of acid-base imbalance, and anti-inflammatory medications for Cl_2_-exposed patients, with no mechanism-based therapeutics available (11, 13). Although several potential therapeutic agents have been tested for efficacy against Cl_2_-induced ALI in different preclinical animal models, none has been approved by regulators (11, 20–22).

The diagnostic and forensic biomarkers of Cl_2_ inhalation injuries are very limited and have been limited to preclinical species. Suspected Cl_2_-exposed victims are tested with nonspecific triage screening tests such as pulse oximetry, electrocardiogram (ECG), complete blood counts (CBC), chemistry panel, arterial blood gas analysis, chest radiography or computed tomography (CT) scanning, ventilation-perfusions scan, pulmonary function testing, and bronchoscopy (23). However, these tests are nonspecific, and clinical signs may overlap with acute respiratory distress syndrome (ARDS) caused by other etiological factors. Therefore, highly sensitive and specific diagnostic biomarkers are needed. Previous in vitro and preclinical in vivo studies showed the potential utility of chlorinated tyrosine adducts (CTAs) [3-chlorotyrosine (Cl-Tyr) and 3,5-dichlorotyrosine (Cl2-Tyr)], chlorinated lipids (2-chloroacids, 2-chloroalcohols, and 2-chloroaldehydes), and phosphatidylglycerol chlorohydrins as forensic biomarkers (24–27). However, these potential biomarkers have not been evaluated in a large animal model that is close to humans in phylogeny.

Due to ethical and feasibility issues surrounding clinical trials of chemical threat agent exposures in humans, regulators such as the United States Food and Drug Administration (US FDA) made provisions to approve therapeutics for human use under the animal rule (21 CFR 314.600) (28). The animal rule requires proof of human safety for a given therapeutic, and demonstration of efficacy in animal models that closely recapitulate the human natural history of the targeted condition, preferably including higher mammalian species. Rodent models (mouse, rat) have been routinely used to screen for candidate therapeutics of Cl_2_-induced ALI; however, these models may not recapitulate human pathophysiology (11, 21, 29, 30). Furthermore, data from a preclinical species that is close to humans in phylogeny improve the confidence of the results. A reproducible large animal model that recapitulates the key features of human Cl_2_-induced ALI/ARDS such as reduced oxygenation and changed respiratory physiological parameters and pulmonary mechanics is urgently needed for testing and regulatory approval of therapeutic candidates. Biomarkers confirming Cl_2_ exposure have not yet been validated in large animal models, complicating exposure dose predictions for forensic analysis after accidents and in chemical weapons fact-finding missions (24, 31–33).

Therefore, we developed a pig translational model of human Cl_2_-induced ALI that enables potential medical countermeasure screening and approval under the US FDA animal rule and validated chlorinated fatty acids (CFAs) and chlorinated tyrosine adducts (CTAs) as potential forensic diagnostic biomarkers of Cl_2_ inhalation exposure.

## MATERIALS AND METHODS

Please see additional details on materials and methods in the Supplemental Material (all Supplemental Material is available at https://doi.org/10.6084/m9.figshare.22901615).

### Preparation of Animals, Cl_2_ Exposure, and Data Collection

**Figure 1.**
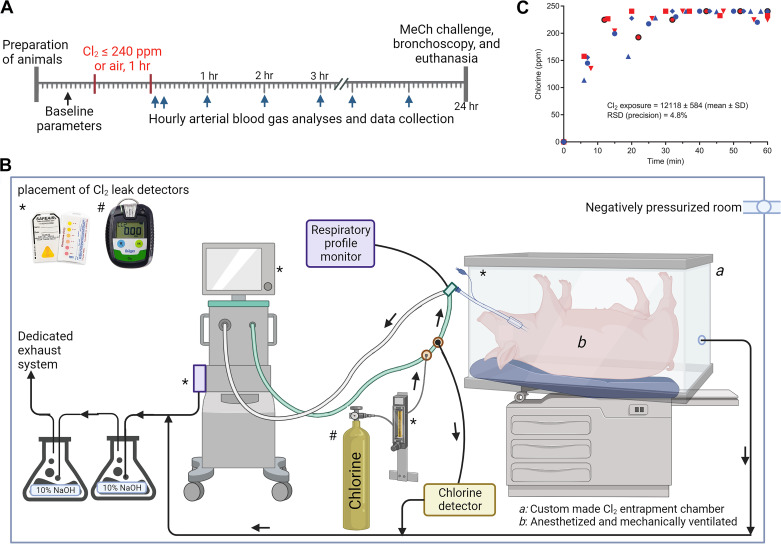
Study design paradigm (*A*), chlorine gas exposure schematic (*B*), and interday precision of chlorine gas exposure (*C*). Anesthetized and mechanically ventilated pigs were exposed to either chlorine gas (Cl_2_) at ≤240 ppm or filtered room air for 1 h. Cl_2_ exposure was monitored every 8–15 min. Arterial blood gas analysis was performed at hourly intervals, and all key oxygenation and respiratory physiological parameters were recorded. At 24-h post Cl_2_ or filtered air exposure, methacholine (MeCh) airway challenge was performed, bronchoalveolar lavage fluid was collected with the aid of bronchoscopy, and pigs were then euthanized to conduct gross necropsy and sample collection. [Images created with a licensed version of BioRender.com].

Male and female Yorkshire pigs (*n* = 14; castrated males and intact females; 30–40 kg) were acclimated at least 48 h after delivery. Pigs were housed in an Association for Assessment and Accreditation of Laboratory Animal Care International-accredited facility at Duke University School of Medicine, Durham, NC, USA. The Duke University Institutional Animal Care and Use Committee approved the procedures in this study. Animals were anesthetized and mechanically ventilated. After placement of surgical instrumentation and acquisition of baseline data (respiratory and cardiovascular physiological parameters, and arterial blood gas analysis), animals were randomized to Cl_2_ or filtered room air exposure (*n* = 6/air group; 8/Cl_2_ group). (Two pigs died during or a few hours after Cl_2_ exposure; therefore, data were excluded from those two pigs and presented for 6/group.) Cl_2_ was delivered via the inspiratory limb of the mechanical ventilator circuit and monitored with a Cl_2_ detector (Porta Sense II Gas Leak Detector, AFC International, Inc., DeMotte, IN) at 8- to 15-min intervals to ensure delivery of ≤240 ppm for 1 h. Cl_2_ exposure occurred in a negatively pressurized room with adequate ventilation and safety monitoring for leak detection in multiple locations. Figure 1, *A* and *B*, shows the study paradigm and the schematic of the Cl_2_ exposure system for pigs.

We collected complete blood counts (CBCs) and serum chemistry panel values at baseline and at 24-h postexposure. The key respiratory physiological parameters were recorded and arterial blood gas analysis was performed at 5- and 10-min postexposure, and then subsequently at 1 h intervals until euthanasia at 24-h postexposure. Oxygenation parameters, i.e., the ratio of the partial pressure of oxygen in arterial blood to the fraction of inhaled oxygen (${\text{PaO}}_{2}$/$\text{F}\text{I}{\text{O}}_{2}$), oxygenation index, saturation of oxygen in hemoglobin ($\text{Sp}{\text{O}}_{2}$), and alveolar-arterial (A-a) gradient, were measured. Respiratory parameters, such as dynamic compliance (*C*_dyn_), airway resistance (Raw), and peak inspiratory pressure (PIP), were assessed at hourly intervals. At 24-h post Cl_2_ or air exposure, airway reactivity was measured by challenging pigs with increasing concentrations of methacholine hydrochloride (MeCh) (0, 0.5, 1, 2, 4, 8, 16, and 32 mg/mL; Sigma-Aldrich, St. Louis, MO) via an Aeroneb nebulizer (Aerogen Ltd., Ireland). Bronchoscopy was performed to collect bronchoalveolar lavage fluid (BALF) just before euthanasia, and total and differential leukocyte counts were determined as previously published, with some modifications (21).

At the end of the 24 h, pigs were euthanized following American Veterinary Medical Association (AVMA) guidelines on euthanasia, and lung tissues were collected for histopathology and diagnostic marker analysis (34). A board-certified veterinary pathologist performed blinded histopathological scoring of lung specimens following criteria to assess ALI recommended by the American Thoracic Society, with some modifications (Supplemental Table S1) (35). The lung wet/dry weight ratio was determined as a surrogate marker for pulmonary edema. Proinflammatory cytokine markers such as IL-6 and VEGF were quantified in BALF supernatants, serum samples, and lung tissue homogenates using ELISA (R&D Systems, Minneapolis, MN).

### Chlorinated Fatty Acids in Plasma and Lung Tissues

Free and total (i.e., free + esterified) 2-chlorofatty acid biomarkers in plasma were measured as previously described, by LC/MS following Dole extraction, using 25 µL of plasma spiked with 103.5 fmol of 2-chloro-[d_4_-7,7,8,8]-palmitic acid (2-[d_4_]-ClPA) as the internal standard (36). For the determination of total 2-chlorofatty acids, extracts were subjected to base hydrolysis before LC/MS. For determination in lung tissue, 20 mg of tissue was pulverized and subjected to Bligh-Dyer lipid extraction (37) and further processed and analyzed by LC/MS as aforementioned.

### Chlorinated Tyrosine Adducts in Plasma and Lung Tissues

The biomarkers 3-chlorotyrosine (Cl-Tyr) and 3,5-dichlorotyrosine (Cl2-Tyr) were isolated from the pronase digest of plasma and lung tissue samples of air or Cl_2_-exposed pigs by solid-phase extraction (SPE), separated by reversed-phase HPLC and detected by tandem mass spectrometry (MS-MS), following previously published methods (26, 38).

### Data and Statistical Analysis

**Figure 2.**
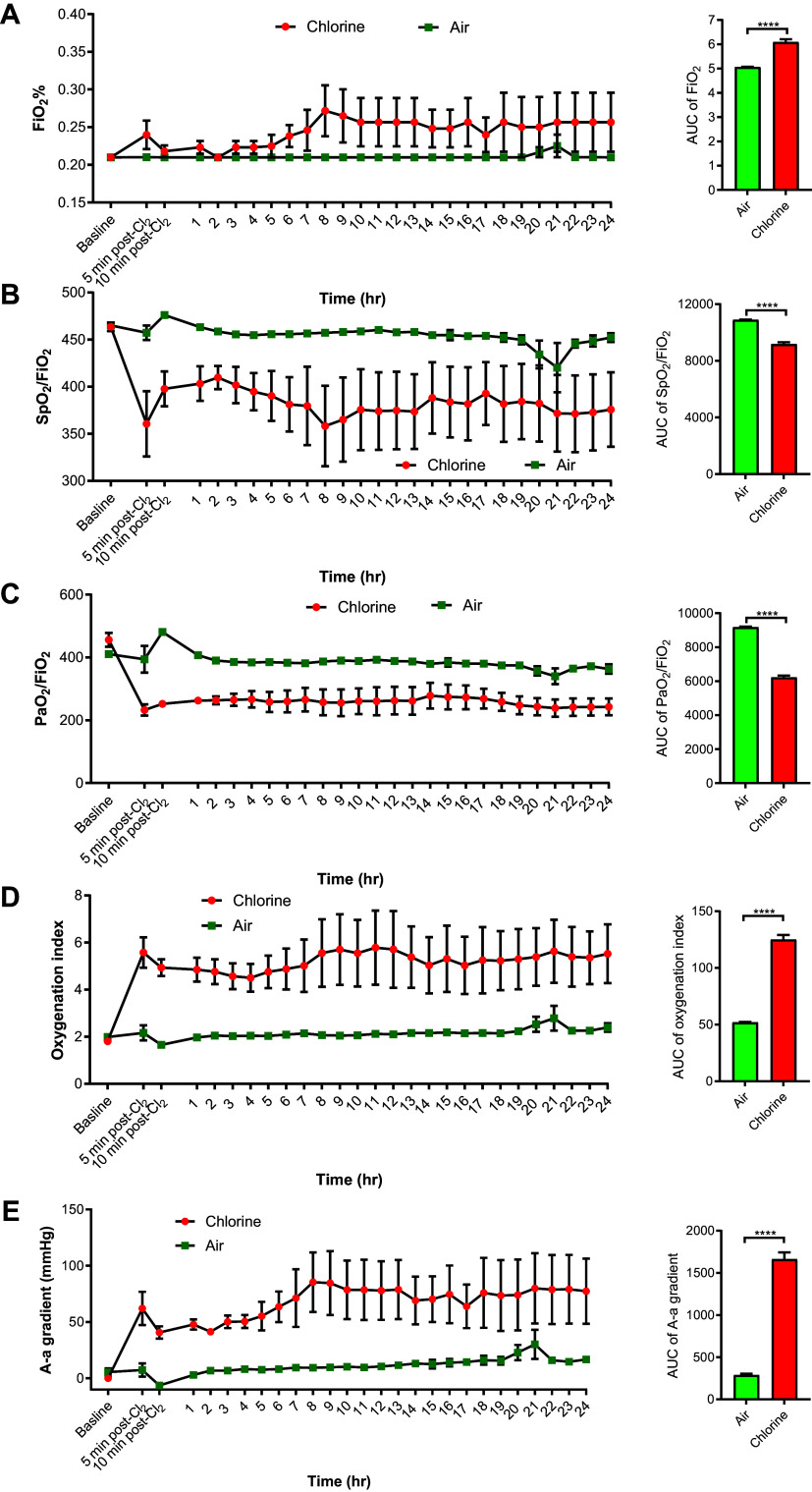
Oxygenation parameters in chlorine-exposed pigs. Anesthetized and mechanically ventilated pigs were exposed to either Cl_2_ at ≤240 ppm or filtered room air for 1 h. The fraction of inhaled oxygen ($\text{F}\text{I}{\text{O}}_{2}$) (*A*), oxygen saturation in hemoglobin normalized by $\text{F}\text{I}{\text{O}}_{2}$ ($\text{Sp}{\text{O}}_{2}$/$\text{F}\text{I}{\text{O}}_{2}$) (*B*), the ratio of partial pressure of oxygen to fraction of inhaled oxygen ($\text{Pa}{\text{O}}_{2}$/$\text{F}\text{I}{\text{O}}_{2}$) (*C*), oxygenation index (*D*), and alveolar-arterial (A-a) gradient (*E*) were measured at hourly intervals. The time course scatterplots show the dynamics of the oxygenation parameters over the 24-h study period. Bar graphs show the area under the curve over the 24-h study period and were compared by unpaired two-tailed *t* test. Data are presented as means ± SEM. *n* = 6/group. ****P < 0.0001.

**Figure 3.**
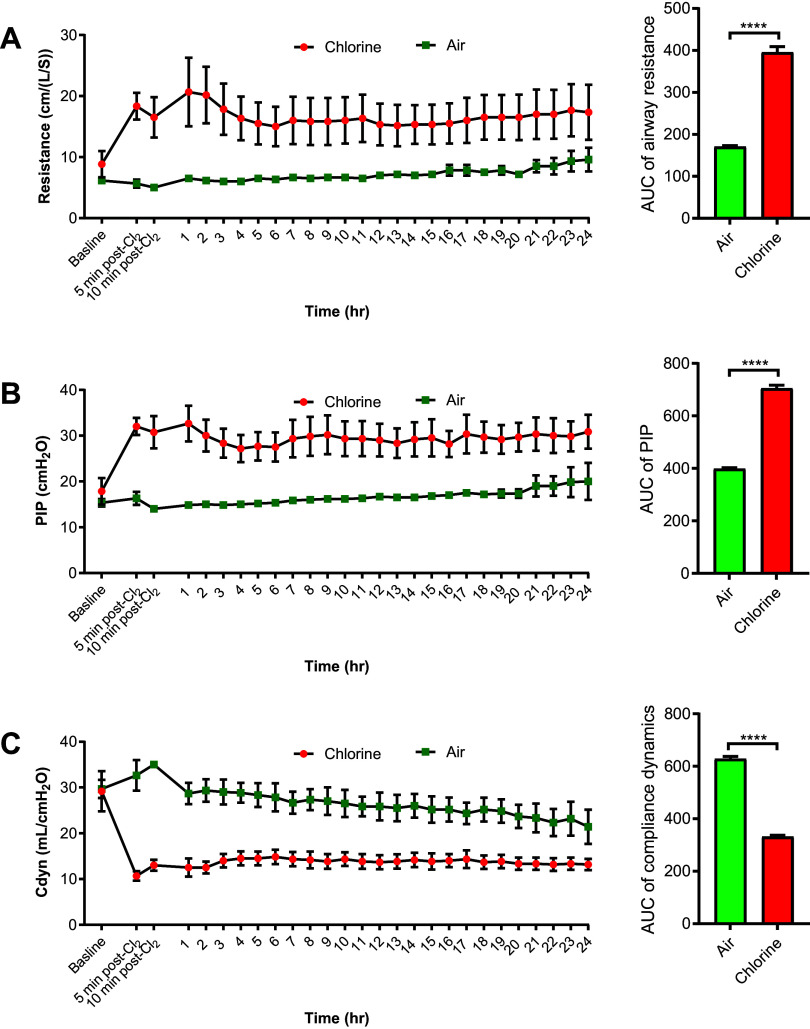
Pulmonary physiological parameters in chlorine-exposed pigs. Anesthetized and mechanically ventilated pigs were exposed to either Cl_2_ at ≤240 ppm or filtered room air for 1 h. Airway resistance (*A*), peak inspiratory pressure (PIP) (*B*), and dynamic compliance (*C*_dyn_) (*C*) were measured at hourly intervals. The time course scatterplots show trends in pulmonary physiological parameters over the 24-h study period. Bar graphs show the area under the curve over the 24-h study period and were compared by unpaired two-tailed *t* test. Data are presented as means ± SEM. *n* = 6/group. ****P < 0.0001.

**Figure 4.**
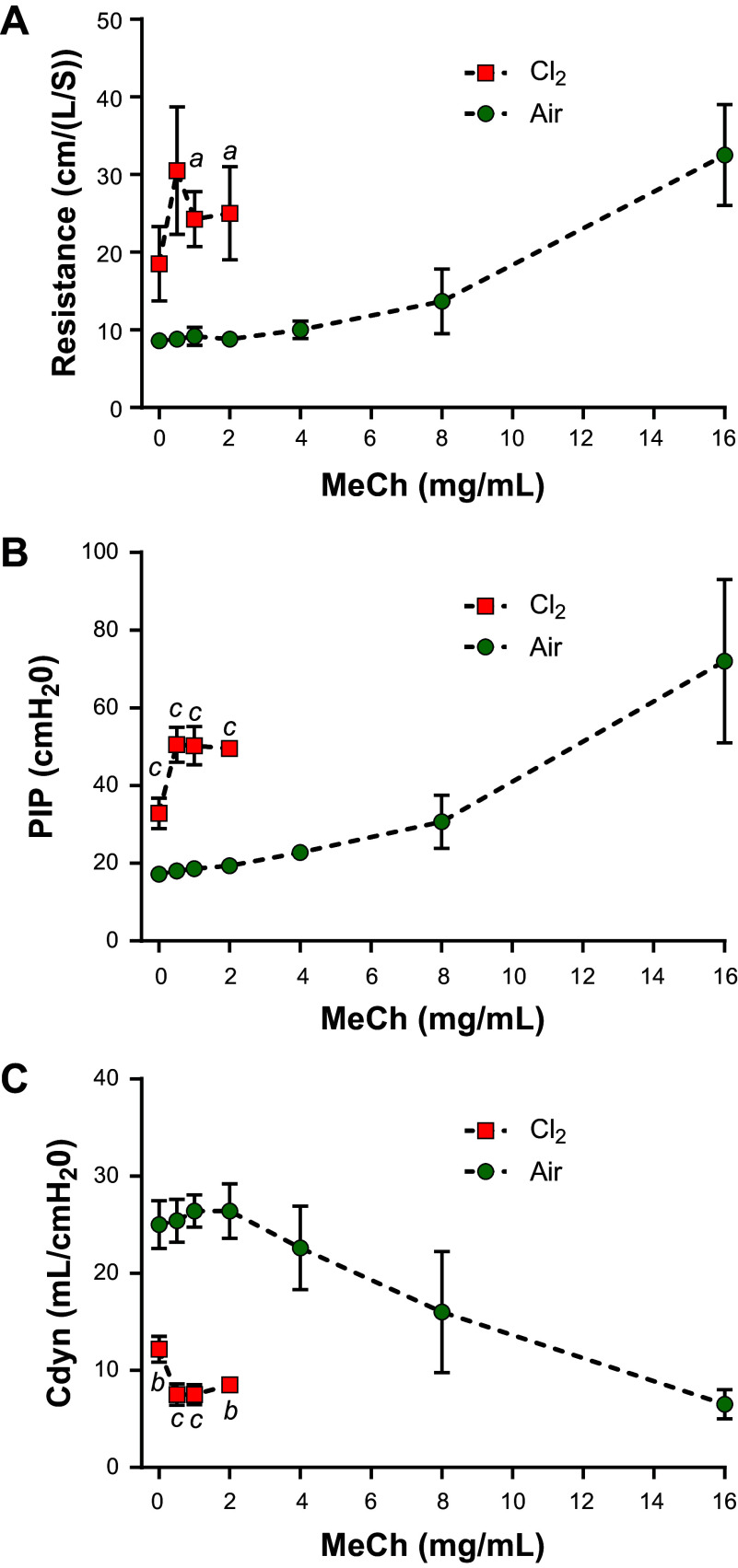
Pulmonary mechanics in chlorine-exposed pigs. Methacholine airway challenge was conducted to assess airway mechanics in Cl_2_- or air-exposed pigs. Airway resistance (*A*), peak inspiratory pressure (PIP) (*B*), and dynamic compliance (*C*_dyn_) (*C*) were measured. Unpaired two-tailed *t* tests were performed between Cl_2_ and air groups at each concentration of methacholine. Data are presented as means ± SEM. *n* = 6/Cl_2_-exposed and 5/air-exposed pigs. ^a^*P* < 0.05; ^b^*P* < 0.01; ^c^*P* < 0.001.

**Figure 5.**
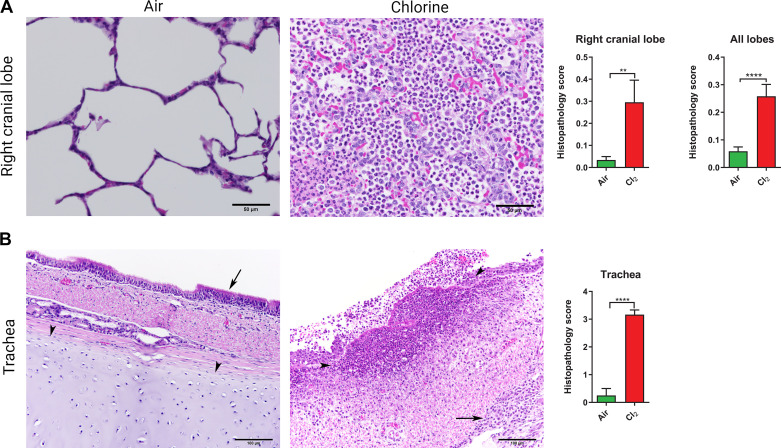
Histopathology of lungs in pigs exposed to chlorine gas. Anesthetized and mechanically ventilated pigs were exposed to either Cl_2_ at ≤240 ppm or filtered room air for 1 h. Histopathological analysis was performed following an official American Thoracic Society (ATS) Workshop Report*:* Features and Measurements of Experimental Acute Lung Injury in Animals (35), with some modifications. *A*: representative images of the right cranial lobe of lungs are presented. In the air group, normal open alveolar architecture of parenchyma is seen without any significant lesions. In the Cl_2_ group, note partial to complete occlusion of the alveolar air spaces by neutrophils and macrophages, thickening of the alveolar septa by similar inflammatory cells, and edema. Some of the alveolar spaces exhibit protein (either fluid or fibrin). The bar graphs show histopathology scores both in the right cranial lobe and in all lobes. *B*: representative tracheal images are presented. In the air group, normal trachea with pseudostratified columnar epithelium (arrows) and uniform lamina and cartilage (arrowheads) is presented. In the Cl_2_ group, the tracheal mucosa is attenuated with the loss of cilia, and multifocal, abrupt ulcers and marked infiltrates by primarily neutrophils (suppuration) are present (outlined by arrowheads). The surface of the mucosa is overlaid by cell debris, and the deeper lamina (arrow) contains infiltrates by lymphocytes, plasma cells, and macrophages. Tracheal ring cartilage is visible in the air-exposed sections, but not in the Cl_2_-exposed sections due to edema and inflammation, although all images were taken at the same magnification. The bar graph shows the histopathology score of the trachea. Bar graph data are presented as means ± SEM. *n* = 6/group. **P ≤ 0.01; ****P < 0.0001. All images are presented at ×40 magnification.

**Figure 6.**
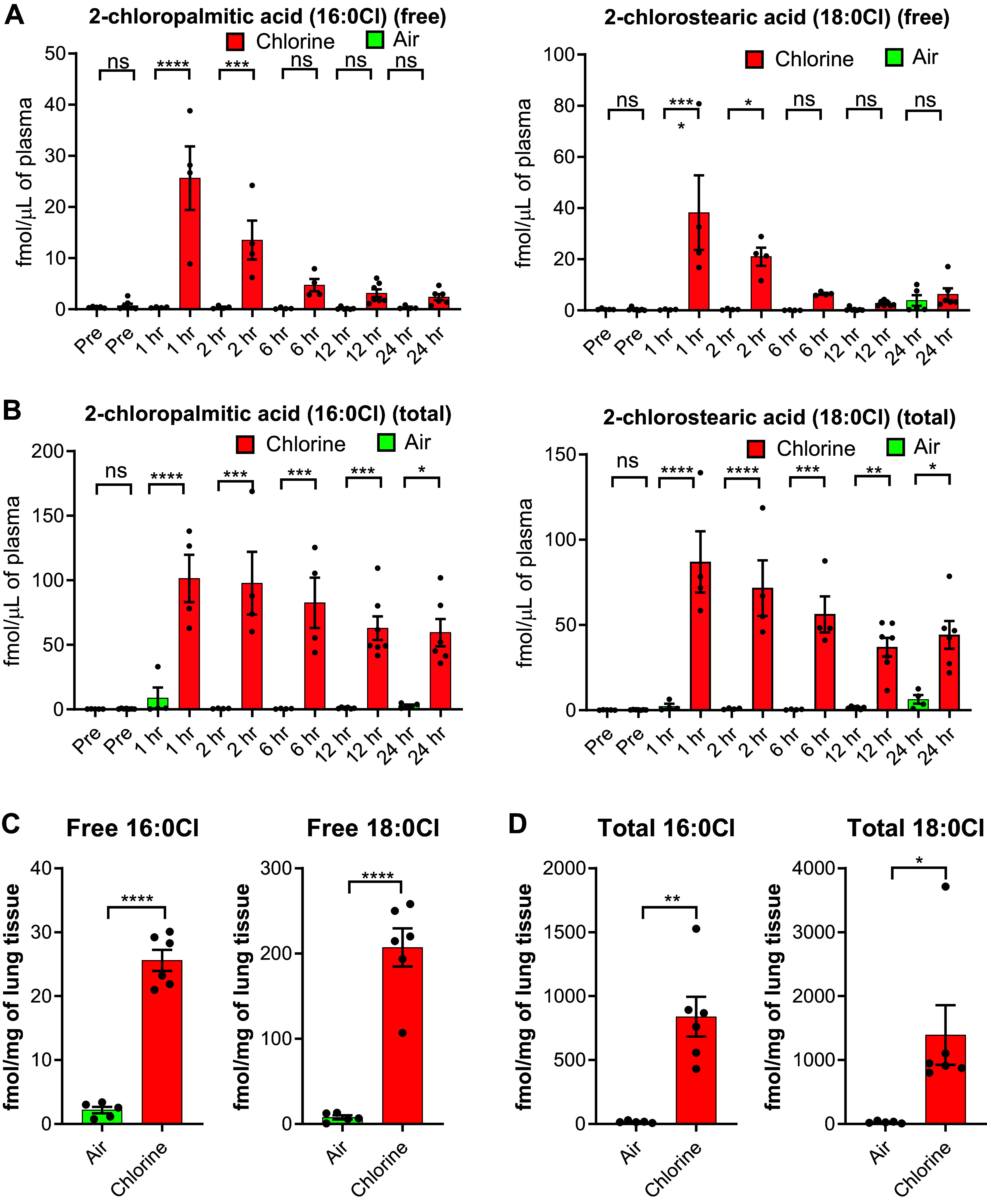
Chlorinated fatty acids in plasma and lung tissues of chlorine-exposed pigs. Free and total (i.e., free + esterified) 2-chlorofatty acids were detected in plasma and lung tissues from both the Cl_2_ and air groups. Free and total fatty acids (2-chloropalmitic acid, 16:0 Cl and 2-chlorostearic acid, 18:0 Cl) were increased in plasma samples that were collected at multiple time points until 24-h post Cl_2_ exposure (*A* and *B*). Both free and total 2-chloropalmitic acid (*C*) and 2-chlorostearic acid (*D*) were significantly increased in lung tissue samples harvested at 24-h post Cl_2_ exposure compared with filtered room air exposure. Red bars represent the Cl_2_ group and green bars represent the air group. Data are presented as means ± SEM. *n* = 6/group. *P ≤ 0.05; **P ≤ 0.01; ***P ≤ 0.001; ****P < 0.0001; ns, not significant.

**Figure 7.**
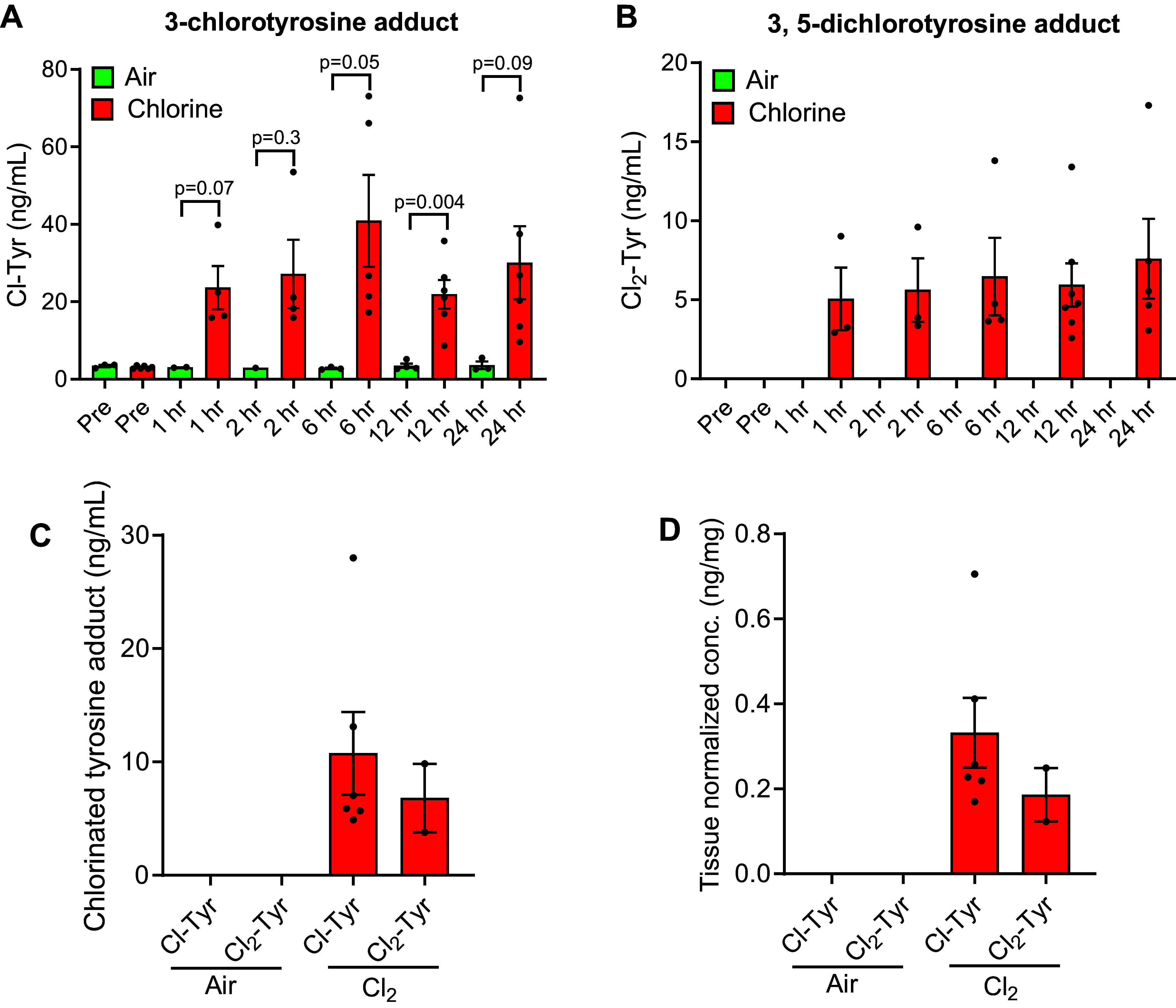
Chlorinated tyrosine adducts in plasma and lung tissues of chlorine-exposed pigs. 3-Chlorotyrosine (Cl-Tyr) (*A*) and 3,5-chlorotyrosine (Cl_2_-Tyr) (*B*) concentrations remained close to lower reportable level (LRL) in plasma samples collected from air-exposed pigs, whereas the concentrations have increased significantly in plasma samples collected from Cl_2_-exposed pigs. In air-exposed animals, the concentrations of Cl-Tyr and Cl_2_-Tyr were nondetectable, whereas were increased by several folds in Cl_2_-exposed animals (*C* and *D*). Data are presented as means ± SEM. *n* = 6/group.

Data are presented as means ± standard error of mean estimate (SEM) in figures and tables. Oxygenation and respiratory physiological parameters are presented as time course scatterplots over 24 h, and bar graphs represent the area under the curve (AUC) from post Cl_2_ or air exposure through 24 h. Unpaired Student’s *t* test (two-tailed) was performed for comparison of means of two groups for statistical analysis unless mentioned (GraphPad Prism v.10.0.3 for Windows, GraphPad Software, San Diego, CA). An α value <0.05 was considered significant. Statistical significance was denoted as *P ≤ 0.05; **P ≤ 0.01; ***P ≤ 0.001; ****P < 0.0001; ns = nonsignificant = *P* > 0.05. Supplemental Table S5 shows details of sample size and statistical tests performed for Figs. 1–7.

## RESULTS

We chose a combination of 240 ppm for the concentration of Cl_2_ and 1 h for the time of exposure to recapitulate human fatal exposure conditions. Figure 1*C* shows interday variability and precision of Cl_2_ exposure. The interday precision of AUC of Cl_2_ exposure was 4.8%.

### Oxygenation Parameters

Cl_2_ exposure resulted in immediate hypoxia and hypoxemia. Pigs in both groups were initially ventilated with 21% oxygen. Later, during and after the end of Cl_2_ exposure, $\text{F}\text{I}{\text{O}}_{2}$ was adjusted to maintain at least 80% $\text{Sp}{\text{O}}_{2}$. Pigs in the Cl_2_ group required higher $\text{F}\text{I}{\text{O}}_{2}$ values (95% confidence intervals [CI] of AUC, 5.6–6.6) compared with the air group (95% CI of AUC, 5.0–5.1) to maintain $\text{Sp}{\text{O}}_{2}$ values above 80% (Fig. 2*A*). $\text{Sp}{\text{O}}_{2}$/$\text{F}\text{I}{\text{O}}_{2}$ values in the Cl_2_ group (95% CI of AUC, 8,580–9,776) were significantly decreased compared with the air group (95% CI, 10,764–10,999; Fig. 2*B*). $\text{Pa}{\text{O}}_{2}$/$\text{F}\text{I}{\text{O}}_{2}$ ratios in the Cl_2_ group (95% CI of AUC, 5,663–6,777) were significantly lower than in the air group (95% CI, 9,013–9,343; Fig. 2*C*). The oxygenation index (OI) was significantly increased in the Cl_2_ group (95% CI of AUC, 105–145) compared with the air group (95% CI, 49.4–54.3; Fig. 2*D*). The alveolar-arterial (A-a) gradient was significantly elevated in the Cl_2_ group (95% CI of AUC, 1,303–2,020) compared with the air group (95% CI, 224–355; Fig. 2*E*).

### Respiratory Mechanical Parameters

In the Cl_2_ group, airway resistance increased significantly when measured after exposure (95% CI of AUC, 332–459) compared with the air group (95% CI, 152–182), staying elevated until the end of the experiment (Fig. 3*A*). Similarly, PIP significantly increased in the Cl_2_ group (95% CI of AUC, 645–764) compared with the air group (95% CI, 378–419; Fig. 3*B*). *C*_dyn_ was significantly decreased in the Cl_2_ group (95% CI of AUC, 306–357) compared with the air group (95% CI, 583–673; Fig. 3*C*).

### Methacholine Airway Challenge

Twenty-four hours postexposure, the Cl_2_-group displayed heightened basal pulmonary resistance compared with the air group. In the Cl_2_ group, airway resistance and PIP were elevated while *C*_dyn_ was decreased at the lowest MeCh concentrations. Cl_2_ group showed early saturated responses with increasing concentrations of MeCh, whereas the air group tolerated higher concentrations of MeCh (Fig. 4, *A–C*).

### Acid-Base Imbalance in Cl_2_-Exposed Pigs

In the Cl_2_ group, immediately after Cl_2_ exposure, pH and HCO_3_^−^ dropped, and partial pressure of carbon dioxide in the arterial blood ($\text{Pa}\text{C}{\text{O}}_{2}$) increased. Due to a compensatory increase in HCO_3_^−^, the pH increased, and then decreased toward the end of the study. However, over 24-h observation, no significant difference in AUCs of pH was noted between the air and Cl_2_ groups. The $\text{Pa}\text{C}{\text{O}}_{2}$ and HCO_3_^−^ were decreased in the Cl_2_-exposed group, whereas lactate values increased (Supplemental Fig. S1). The resulting acid-base imbalance over 24 h suggests respiratory alkalosis with metabolic acidosis.

### BALF Total Cell Counts and Differential Leukocyte Counts, Protein Leak, and Pulmonary Edema

The total BALF leukocyte count was significantly increased in Cl_2_ pigs compared with the air group, consisting mostly of neutrophils (Supplemental Fig. S2, *A* and *B*). BALF protein levels and wet/dry lung weight ratio were significantly increased in the Cl_2_ group compared with the air group (Supplemental Fig. S2, *C* and *D*).

### Proinflammatory Cytokine Markers

IL-6 and VEGF levels trended higher in Cl_2_ pigs in all three biological matrices tested, i.e., BALF, serum, and lung tissue homogenates (Supplemental Fig. S2, *E*–*G*).

### Complete Blood Counts and Serum Chemistry Panels

CBC and serum chemistry panel values are presented in Supplemental Tables S3 and S4, respectively. Platelet counts had significantly decreased in both groups at 24-h postexposure compared with their respective baseline values. Also, the mean platelet count was different between air- and Cl_2_-exposed groups at the 24-h time point. Although other parameters had some trends in CBC, they did not reach statistical significance. Among serum chemistry panel values, there was no significant difference between air- and Cl_2_-exposed groups at the 24-h time point. However, there was a significant difference between values at baseline and the 24-h time point for glucose, cholesterol, and alkaline phosphatase in both air- and Cl_2_-exposed groups.

### Postmortem Examination and Histopathological Analysis

Postmortem gross examination of the lungs revealed diffuse lesions of atelectasis and hemorrhage in the Cl_2_ group, whereas the air group had no detectable abnormal findings. However, caudal atelectatic lesions were found in the lungs of both groups. Such lesions are consistent with mechanical ventilator-induced lung injury (VILI). In pigs exposed to air, histopathological analysis of the lungs revealed normal open alveolar architecture of the parenchyma without any significant lesions. In pigs exposed to Cl_2_, however, lung sections showed partial to complete occlusion of the alveolar air spaces by neutrophils and macrophages as well as thickening of the alveolar septa by similar inflammatory cells and edema, and some of the alveolar spaces exhibited protein (either fluid or fibrin). Histopathological scores were significantly higher in the Cl_2_ group compared with the air group (Fig. 5*A*). In Cl_2_ pigs, the tracheal mucosa was completely disrupted and overlaid with cell debris, whereas histopathological analysis of the trachea from the air group showed normal architecture (Fig. 5*B*).

### Chlorinated Fatty Acids

Free and total (i.e., free + esterified) 2-chlorofatty acids were detected in plasma and lung tissues from both groups. Free fatty acids (2-chloropalmitic acid, 16:0 Cl and 2-chlorostearic acid, 18:0 Cl), which are not esterified to complex lipids, were significantly increased in plasma samples collected within 2 h after Cl_2_ exposure (Fig. 6*A*), whereas total fatty acids (16:0 Cl and 18:0 Cl) were significantly increased in plasma samples that were collected up to 24 h post Cl_2_ exposure compared with correspondingly timed samples from pigs in the air group (Fig. 6*B*). Both free and total 2-chloropalmitic acid and 2-chlorostearic acid were significantly increased in lung tissue harvested at 24 h post Cl_2_ exposure compared with air exposure (Fig. 6, *C* and *D*).

### Chlorinated Tyrosine Adducts

An analytical method to quantify Cl-Tyr and Cl_2_-Tyr in pig lung tissues and plasma was established (Supplemental Table S2 and Supplemental Fig. S3). Although baseline levels of Cl-Tyr were detected in plasma of air-exposed pigs, Cl_2_-Tyr was absent. Both adducts were strongly increased in plasma of Cl_2_-exposed pigs, with levels remaining constant over 24 h. Adducts were undetectable in lung tissue of air-exposed pigs, with levels strongly increased in Cl_2_-exposed pigs [Cl-Tyr levels increased to 10.8 ± 3.6 (means ± SEM) ng/mL and Cl_2_-Tyr levels increased to 6.8 ± 3 (means ± SEM) ng/mL] (Fig. 7).

## DISCUSSION

In this study, we developed a translational model of ALI in pigs that will meet US FDA animal rules (28). We chose the pig as a translational model to study chemically induced ALI in humans due to their anatomical and physiological similarities (39, 40). Previous studies have laid the foundation for our animal model development study (41–48). Our model overcomes several limitations of models described in the literature (41–48). In these previous studies, pigs were exposed to Cl_2_ at concentrations ranging from 100–140 ppm for 10 min [CT (concentration-time product) = 1,000–1,400 ppm·min] to 400 ppm for 15–20 min (CT = 6.000–8,000 ppm·min). In the present model, the AUC of the concentration-time curve of Cl_2_ exposure was 12,118 ± 584 ppm·min (means ± SD). Our Cl_2_ exposure conditions were equivalent to predicted fatal exposures in humans (>400 ppm of Cl_2_ for 30 min, that is, CT = 12,000 ppm·min) (14).

The present study observed the pathophysiology of Cl_2_-induced ALI over 24 h, a longer time span than previous studies observing for 5- to 12-h postexposure. Positive end-expiratory pressure (PEEP) was not used in the previously published models, whereas we applied a 5-cmH_2_O PEEP to prevent the collapse of alveoli during exhalation, and to improve alveolar recruitment and oxygenation capacity (42, 43, 45, 47). Although the accepted range of tidal volume (VT) is 6–8 mL/kg, previous studies used VT outside the accepted volumes, ranging from 8 to 10 mL/kg (45), 10 mL/kg (47), 12 mL/kg (43), and 15–20 mL/kg (42), whereas 7 mL/kg was used here. Low tidal volumes help in reducing the likelihood of VILI, which is a confounding factor in inhalation injury studies. Watkins et al. (48) developed a spontaneously breathing chlorine inhalation swine model, which can avoid potential VILI. We maintained pigs on pressure-regulated volume control and assist control (PRVC A/C) mode of mechanical ventilation. In PRVC mode, the preset tidal volume is maintained by adapting to the changing compliance of the lungs to adjust inspiratory time and pressure. As A/C is also engaged in this setting, respiration is completely under the control of the mechanical ventilator and ensures that pigs will not initiate any breaths.

### Oxygenation Parameters

Exposure to Cl_2_ resulted in immediate hypoxia and hypoxemia. The *P*/*F* ratio ($\text{Pa}{\text{O}}_{2}$/$\text{F}\text{I}{\text{O}}_{2}$), is generally used in critical care medicine to assess oxygenation use in patients. *P*/*F* ≤ 300 and ≤200 mmHg are signs of ALI and ARDS, respectively (49). *P*/*F* ratios decreased below 300 in our Cl_2_-exposed pigs at the end of the exposure and remained the same or decreased further over the following 24 h, indicating ALI. The oxygenation index (OI) is recognized as a more sensitive marker than the P/F ratio for assessing oxygenation and injury because lung mechanics (mean airway pressure) are considered in the calculation. Lower OI values indicate better use of inhaled oxygen and are desirable. In the present studies, Cl_2_-exposed pigs had elevated OI values, indicating poor use of inhaled oxygen. $\text{Sp}{\text{O}}_{2}$ indicates oxygen saturation levels in hemoglobin. We started all pigs at room air ($\text{F}\text{I}{\text{O}}_{2}$ of 21%); after the exposure, we adjusted $\text{F}\text{I}{\text{O}}_{2}$ to maintain at least 80% $\text{Sp}{\text{O}}_{2}$. The Cl_2_ group required substantially higher $\text{F}\text{I}{\text{O}}_{2}$ to maintain at least 80% $\text{Sp}{\text{O}}_{2}$ compared with the air group. As higher $\text{F}\text{I}{\text{O}}_{2}$ values can result in the production of more reactive oxygen species, we tried to maintain a modest value of at least 80% $\text{Sp}{\text{O}}_{2}$. Targeting a higher $\text{Sp}{\text{O}}_{2}$ value by increasing $\text{F}\text{I}{\text{O}}_{2}$ might be useful, but no controlled studies are available to support this idea, and supplementing higher $\text{F}\text{I}{\text{O}}_{2}$ value has been linked to worsening ALI (50). The A-a gradient as a measure of effective oxygen transfer from the alveoli to the blood was elevated in the Cl_2_ group compared with the air group, suggesting a defect in diffusion and a ventilation-perfusion mismatch. This is not a surprising finding, as previous studies and our findings show that alveoli are damaged with Cl_2_ exposure (21, 48).

### Respiratory Physiological Parameters

Airway resistance and PIP increased significantly in Cl_2_-exposed pigs. *C*_dyn_ decreased rapidly following Cl_2_ exposure. This rapid decrease in *C*_dyn_ may be due to increased resistance to airflow because of airway injury, resulting in regional trapping of air with hyperinflation of lung regions distal to the site of bronchoconstriction. The collapse of pulmonary parenchyma and increase in interstitial fluids may also decrease *C*_dyn_ at later stages of ALI (43, 51). In the MeCh airway challenge test, airway hyperreactivity was readily observed even at the lowest MeCh challenge concentration in Cl_2_-exposed animals, whereas air-exposed animals tolerated all tested doses of MeCh. Furthermore, there was a clear difference in baseline pulmonary mechanics between the two groups before the administration of the first dose of MeCh, which is reminiscent of the increase in baseline pulmonary resistance in human ALI and ARDS, and not commonly observed in rodent models (10, 21, 52).

### Proinflammatory Cytokine Markers

Proinflammatory cytokine markers, such as IL-6 and VEGF, trended higher in the Cl_2_ group compared with air-exposed animals. IL-6 levels were consistently trended higher after Cl_2_ exposure in all three biological matrices tested. Therefore, IL-6 may be useful as a potential proinflammatory cytokine marker to assess disease progression as well as therapeutic efficacy of drug candidates.

### CBC, Chemistry Panel, and Acid-Base Imbalance

As there are no published references for CBC and serum chemistry in a pig model of Cl_2_ inhalation injury, the data presented here will serve as a reference for future work. Although baseline variability was noted in platelet counts in air- and Cl_2_-exposed animals, thrombocytopenia (decreased platelet count) was higher in Cl_2_-exposed animals (52%) compared with air-exposed animals (38%). Platelets perform a variety of tasks in addition to hemostasis, such as modulation of the innate immune system (53, 54). Platelet-depletion studies in animal models have revealed specific roles for platelets in the development of ARDS. Furthermore, thrombocytopenia was associated with increased mortality in patients who developed ARDS but not in those who did not develop ARDS. Therefore, thrombocytopenia is one of the underlying major pathophysiological events that contribute to ARDS (54–58). However, additional studies are warranted to further investigate the role of thrombocytopenia in Cl_2_-induced ALI.

Previous clinical case reports and preclinical studies suggested that exposure to Cl_2_ induced respiratory acidosis. Furthermore, intravenous or nebulized sodium bicarbonate (NaHCO_3_) has been used as a therapeutic agent to correct the acid-base imbalance. However, the evidence for this is mostly anecdotal, or at best, limited data are available to support this treatment (14, 59). In the current study, we noted an evolution of the acid-base status of pigs exposed to Cl_2_ from an initial combined respiratory and metabolic acidosis immediately after Cl_2_ exposure to a mixed respiratory alkalosis and metabolic acidosis by 24-h postexposure. The increased lactate levels in Cl_2_-exposed pigs clearly suggest hypoxia and injury. Elevation of lactate levels was more pronounced in the first half of the study observation time and then the levels remained constantly higher in Cl_2_-exposed animals compared with air-exposed animals. The anesthesia protocol and mechanical ventilation may have had some influence on the observed acid-base imbalance. The lack of published normal values for arterial blood gas analysis in pigs under our anesthetic/analgesic regimen also makes it difficult to compare with human values. Therefore, the data should be interpreted with caution. Together, these findings suggest that generalized treatment of Cl_2_ victims with NaHCO_3_ may at best be used as supportive therapy to manage acid-base imbalance.

### Effects on Cardiovascular Function

In the course of our study, one pig reached humane endpoints during Cl_2_ exposure requiring euthanasia, and another pig requiring euthanasia 3–4 h after Cl_2_ exposure. Both pigs displayed progressive nonresponsive hypotension, decreased heart rate, and hypoxemia. Cardiopulmonary resuscitation efforts with 100% oxygen supplementation, epinephrine injection, and defibrillation were not successful. A postmortem examination revealed that both pigs had preexisting left ventricular hypertrophy (right-to-left ventricular wall thickness ratio ≥ 1:5). Previous studies showed cardiac ventricular hypertrophy in pigs, depending on the birth season, breed, sex, and sire family (60, 61). Nevertheless, this suggests that Cl_2_ exposure in the context of preexisting cardiac dysfunction can result in increased morbidity and mortality. Cl_2_ exposure decreases cardiac output probably due to right ventricular failure secondary to pulmonary vasoconstriction (43). Often, severe right ventricular failure results in death, particularly in patients with preexisting left ventricular failure or left ventricular hypertrophy. Limited animal studies and isolated reports in humans exposed to Cl_2_ suggest detrimental effects on the cardiovascular system (62). Additional detailed studies on cardiovascular effects are warranted. Therefore, we recommend the evaluation of cardiovascular function before initiating Cl_2_ exposure studies in pigs to screen for any potential cardiac anomalies.

### Postmortem Lung Gross Examination and Histopathology

A gross examination of the lungs revealed diffuse atelectatic lesions in the diaphragmatic lobes of both groups. These findings are consistent with the lesions commonly seen in animals restrained in dorsal recumbency (supine positioning) and mechanically ventilated. In this study, animals were mechanically ventilated in the supine position, which is one of the limitations of the study. Prone positioning was shown to be advantageous over supine positioning in both human studies and translational animal models (43, 63–65). Future studies are warranted to prospectively study the advantages of prone positioning and positional maneuvers in Cl_2_-exposed pigs. The lung tissues of Cl_2_-exposed animals exhibited all three key features of human ALI: neutrophilic alveolitis, deposition of hyaline membranes, and formation of microthrombi. Therefore, this animal model may serve as a model for additional forms of ALI in humans (35, 40).

### CFAs and CTAs as Biomarkers of Cl_2_ Exposure

The use of chemical warfare agents such as Cl_2_ is often disputed (1, 2). Therefore, the availability of robust biomarkers is essential for diagnostics and forensic detection. The current literature on biomarkers of Cl_2_ exposures is limited to rodent models and in vitro studies, identifying CFAs and CTAs as potential markers (24, 26, 31–33, 66). Chlorinated fatty acids are produced when Cl_2_ or hypochlorite reacts with plasmalogen. In the present study, free and total CFAs (2-chloropalmititc acid and 2-chlorostearic acid) were significantly increased in both plasma and lung tissues collected from the Cl_2_ group, suggesting that they may be used as diagnostic markers. Both markers were readily detectable immediately after Cl_2_ exposure. Although levels of free CFAs declined over time, total CFAs (free + bound) remained elevated throughout the 24-h observation period, likely due to an increasing amount of bound CFAs. We speculate that CFA levels likely remain elevated until 72-h postexposure or beyond, as observed in rodent studies (32). Recently, these CFAs have been detected in plasma samples of victims of a Cl_2_ accident (66), further validating their utility as forensic biomarkers. In a recent swine study, palmitoyl-oleoyl phosphatidylglycerol chlorohydrin (POPG-HOCl) and the lipid derivative oleoyl ethanolamide chlorohydrin (OEA-HOCl) were shown to increase in BALF samples collected from spontaneously breathing pigs after Cl_2_ exposure (67). Both POPG-HOCl and OEA-HOCl levels correlated with inhaled doses of Cl_2_. Such correlations are impactful for the assessment of the severity of injury after Cl_2_ inhalation. However, their study was limited to BALF samples. It will not be feasible in a mass casualty situation to collect BALF samples readily due to technical and time-sensitive matters.

Previously, chlorinated tyrosine adducts, CTAs (Cl-Tyr and Cl_2_-Tyr), have been detected in tissue samples from Cl_2_-exposed mice, and in Cl_2_-exposed human blood samples under in vitro conditions (26, 38). Although undetectable or remaining below lower reportable level (LRL, 2.5 mg/mL) in air-exposed control pigs, Cl-Tyr and Cl_2_-Tyr were strongly elevated in plasma and lung tissue samples collected from Cl_2_-exposed pigs immediately after exposure until the end of observation after 24 h (38). These observations confirm that both marker classes, initially observed in rodents, are also detectable in plasma and lung tissues of Cl_2_-exposed large animals, strongly supporting their universal utility as biomarkers of Cl_2_ exposure. As pig models are routinely used for studying human cardiopulmonary diseases, identification of these biomarkers in the pig Cl_2_ inhalation model paves the way for further investigation. For forensic investigations, the persistence of CFAs and CTAs in plasma samples 24-h postexposure and beyond is advantageous, paired with the ease of sample collection at multiple time points. Detection of CFAs and CTAs in lung tissue samples is also highly relevant for forensics, establishing that victims were indeed exposed to Cl_2_. Controlled studies in pigs varying Cl_2_ levels and exposure time with subsequent marker measurements may allow the establishment of models for forensic estimation of human exposures essential after accidents and suspected chemical weapons use.

### Clinical Applicability

The model developed here represents several features of humans exposed to fatal concentrations of Cl_2_, including injury following oropharyngeal inhalation. This feature is poorly represented in rodent models since rodents are obligate nasal breathers. Diffusion modeling studies at low Cl_2_ concentrations suggest that ∼90% of inhaled Cl_2_ will be scrubbed in the nasal passages and pharynx, and that only 10% of inhaled Cl_2_ reaches beyond the hypopharynx (68). The actual pathophysiological course of human victims exposed to Cl_2_ is highly variable depending on the concentration of the gas, duration of exposure, and distance of the individual victim from the locus of the source (69). A human exposed to Cl_2_ may experience rapid nasal obstruction and may often attempt to escape from the exposure, likely breathing predominantly through the oropharynx. In our model, pigs were exposed to Cl_2_ via an endotracheal tube not only to ensure homogeneous exposure but also to replicate human oropharyngeal Cl_2_ exposure.

### Limitations of the Study

In this study, observation time was limited to 24 h. However, in the continuum of this work, it would be worthwhile to extubate the animals after titrating Cl_2_ exposure conditions and monitor them over an extended time, at least up to 2 months. In our current Cl_2_ exposure conditions (≤240 ppm for 60 min), extubation may not be feasible, as Cl_2_-exposed pigs may require mechanical ventilator support and supplemental oxygen to ensure adequate tissue oxygenation. Therefore, further optimization of exposure conditions that result in a less severe injury model while still meeting ALI or ARDS criteria is desired. Also, animals under continued mechanical ventilation and anesthesia fail to exhibit their natural physiological protective reflexes such as cough, apnea, and shallow breathing (43). Therefore, studies are warranted to understand the long-term pathophysiology of Cl_2_-exposed and extubated pigs.

### Conclusions

We established a pig model of Cl_2_ inhalation lung injury that recapitulated the pathophysiological course of Cl_2_ lung injury in human exposure victims. Chlorofatty acids and chlorinated proteins were identified as biomarkers of Cl_2_ exposure. This model can serve as an accurate translational model of human Cl_2_-induced ALI for screening and developing chemical exposure countermeasures under the US FDA animal rule. The chlorofatty acids and chlorinated proteins that we identified may support forensic analysis after accidents and suspected chemical weapons use.

## DATA AVAILABILITY

Data will be made available upon reasonable request.

## SUPPLEMENTAL DATA

10.6084/m9.figshare.22901615 Supplemental Tables S1–S5 and Supplemental Figs. S1–S3: https://doi.org/10.6084/m9.figshare.22901615.

## GRANTS

S.A. and S.E.J. are supported by Grants U01ES030672, 1R21ES030331-01A1, and R01ES034387 of the NIH Countermeasures Against Chemical Threats (CounterACT) program. D.A.F. was supported by National Institutes of Health Grant R01GM115553.

## DISCLAIMERS

The content is solely the responsibility of the authors and does not necessarily represent the views of the National Institutes of Health or Food and Drug Administration.

## DISCLOSURES

No conflicts of interest, financial or otherwise, declared by the authors.

## AUTHOR CONTRIBUTIONS

S.A., M.A.G., and S.E.J. conceived and designed research; S.A., M.A.G., C.J.A., K.A.S., B.G.P., B.S.C., J.Q.-G., and J.W.P. performed experiments; S.A., C.J.A., K.A.S., B.G.P., B.S.C., J.Q.-G., J.W.P., D.A.F., and T.A.B. analyzed data; S.A., M.A.G., D.A.F., R.P.P., T.A.B., M.D.G., and S.E.J. interpreted results of experiments; S.A. prepared figures; S.A. and T.A.B. drafted manuscript; S.A., M.A.G., and S.E.J. edited and revised manuscript; S.A., M.A.G., D.A.F., R.P.P., T.A.B., M.D.G., and S.E.J. approved final version of manuscript.
